# Compound climate extremes driving recent sub-continental tree mortality in northern Australia have no precedent in recent centuries

**DOI:** 10.1038/s41598-021-97762-x

**Published:** 2021-09-15

**Authors:** Kathryn J. Allen, Danielle C. Verdon-Kidd, James Z. Sippo, Patrick J. Baker

**Affiliations:** 1 School of Geography and Spatial Science, University of Tasmania, Sandy Bay, 7005 Australia; 2 School of Ecosystem and Forest Sciences, University of Melbourne, Richmond, 3121 Australia; 3 ARC Centre for Australian Biodiversity and Heritage, UNSW Node, Sydney, 2052 Australia; 4 School of Environmental and Life Sciences, University of Newcastle, Callaghan, 2308 Australia; 5 Faculty of Science and Engineering, Southern Cross University, Lismore, 2480 Australia

**Keywords:** Climate sciences, Environmental sciences

## Abstract

Compound climate extremes (CCEs) can have significant and persistent environmental impacts on ecosystems. However, knowledge of the occurrence of CCEs beyond the past ~ 50 years, and hence their ecological impacts, is limited. Here, we place the widespread 2015–16 mangrove dieback and the more recent 2020 inland native forest dieback events in northern Australia into a longer historical context using locally relevant palaeoclimate records. Over recent centuries, multiple occurrences of analogous antecedent and coincident climate conditions associated with the mangrove dieback event were identified in this compilation. However, rising sea level—a key antecedent condition—over the three decades prior to the mangrove dieback is unprecedented in the past 220 years. Similarly, dieback in inland forests and savannas was associated with a multi-decadal wetting trend followed by the longest and most intense drought conditions of the past 250 years, coupled with rising temperatures. While many ecological communities may have experienced CCEs in past centuries, the addition of new environmental stressors associated with varying aspects of global change may exceed their thresholds of resilience. Palaeoclimate compilations provide the much-needed longer term context to better assess frequency and changes in some types of CCEs and their environmental impacts.

## Introduction

Compound climate extremes (CCEs) have significant societal and environmental impacts and cascading effects^1–3^. Four distinct forms of CCEs can be distinguished^3^: (1) pre-conditioned, in which prior conditions accentuate hazard impacts; (2) multivariate, associated with multiple drivers; (3) temporally compounding, in which the impacts of successive events accumulate non-linearly; and (4) spatially compounding, where impacts occur in multiple locations simultaneously. The literature on the impacts of CCEs, however, is heavily focused around human populations, agriculture and engineering infrastructure e.g.,^2,4,5^. It is also significantly limited in temporal scope (often only the past 50–60 years), which is particularly significant if CCEs are rare. Here we focus on two instances of ecological impacts of CCEs and put these in a longer term context by examining a series of locally relevant palaeoclimate records.

In recent years the forests of northern Australia have experienced two massive dieback episodes linked to CCEs. In 2015–16 mangrove forests primarily dominated by *Avicennia marina subsp. Eucalyptifolia* across the eastern shores of the Gulf of Carpentaria, in Kakadu National Park, and the Ningaloo coast died over 1500 square kilometres^6,7^. Then in 2020 inland forests and savannas in the north of the Northern Territory of Australia experienced rapid widespread tree dieback. Both the tropical mangrove forests and tropical savannas have been declared collapsing ecosystems^8^. Both events were associated with pre-conditioned, and in the case of the mangrove dieback, multivariate CCEs^3^.

**Figure 2:**
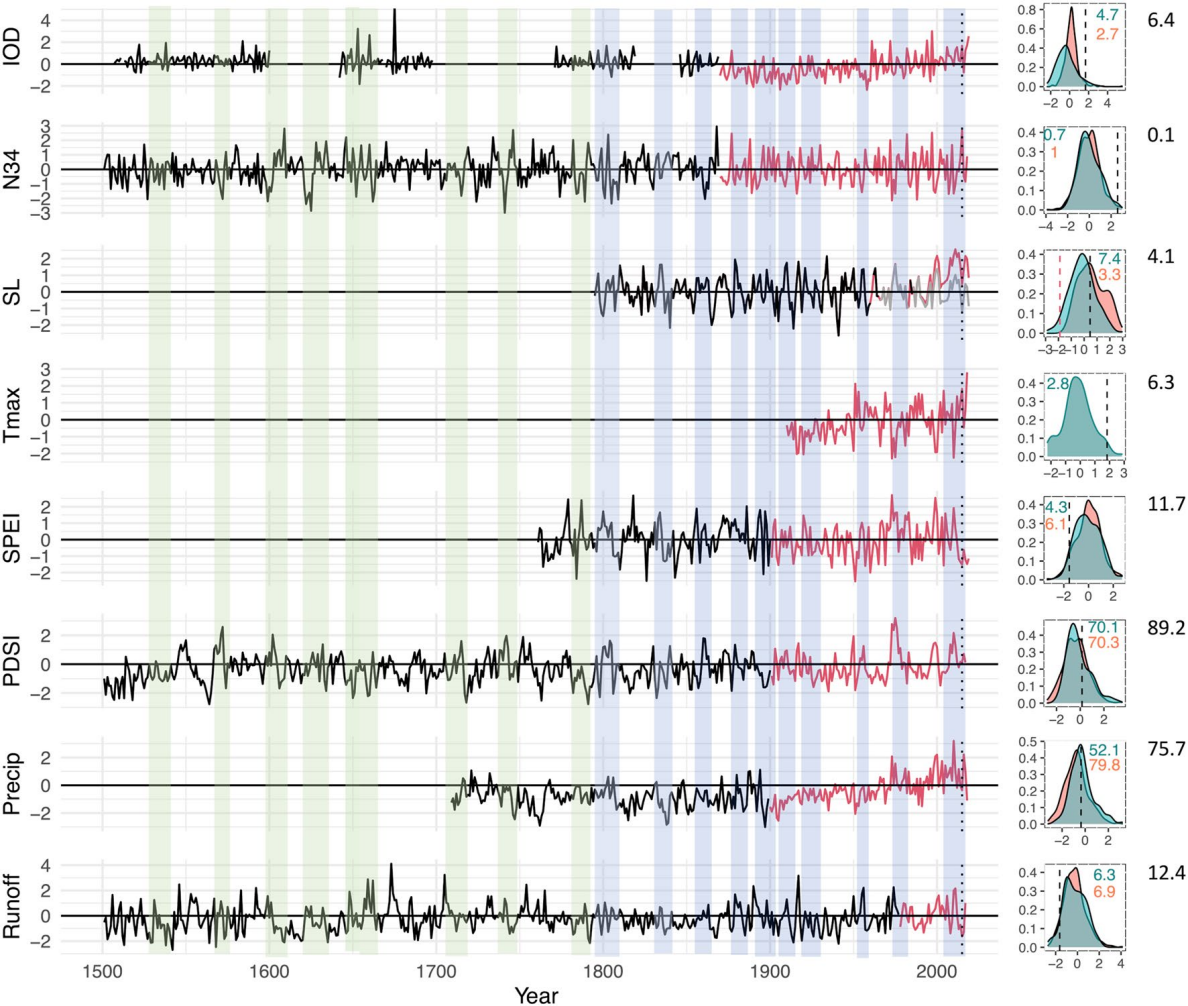
Left panel: Time series (z-scores relative to the 1961–90 period) of reconstructed and instrumental indices (IOD, Niño3.4) and climate variables (Sea level, maximum temperature; SPEI3, PDSI, precipitation (AWAP data); Alligator River run off) relevant for northern Australia. For sea level, red line is the non-detrended sea level data while grey line is the detrended data. Right panel: associated probability distributions, red is for reconstructed series, and teal for instrumental data. Vertical black lines show where in the distribution the 2015–16 value fell. Red line in sea level distribution shows value for 2015–16 once data detrended, black line is for non-detrended data. Numbers in upper left or right relate to the empirical probability (as percentages) of experiencing an event in that variable at least as extreme as that in 2015. Colours match the respective distributions. For sea level, figures are based on detrended sea level data.

**Figure 3:**
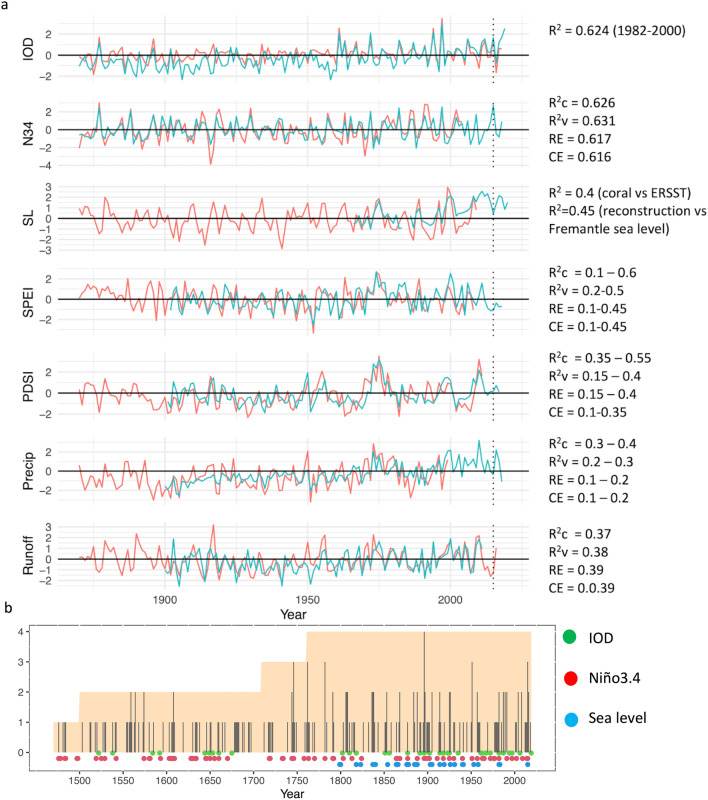
(**a**) Comparison of reconstructed parameters and instrumental data. Reconstructions are red and instrumental data in teal. Statistics next to each plot have been drawn from the original publications (Table 1). Those for the runoff reconstruction have been averaged across the early and late, and (for R^2^c), the whole calibration periods. Note that the runoff reconstruction based on proxies as described in Verdon-Kidd et al. (2017) extends only to 1975 after which it is based solely on streamflow simulated from rainfall, hence the almost perfect fit from 1976 to 2011. (**b**) The number of hydroclimate reconstructions that record single year events. Also shown are those years for which each of the IOD, Nino 3.4 and sea level data shows an event. Background shading indicates number of hydroclimate proxies available for each year.

**Figure 4:**
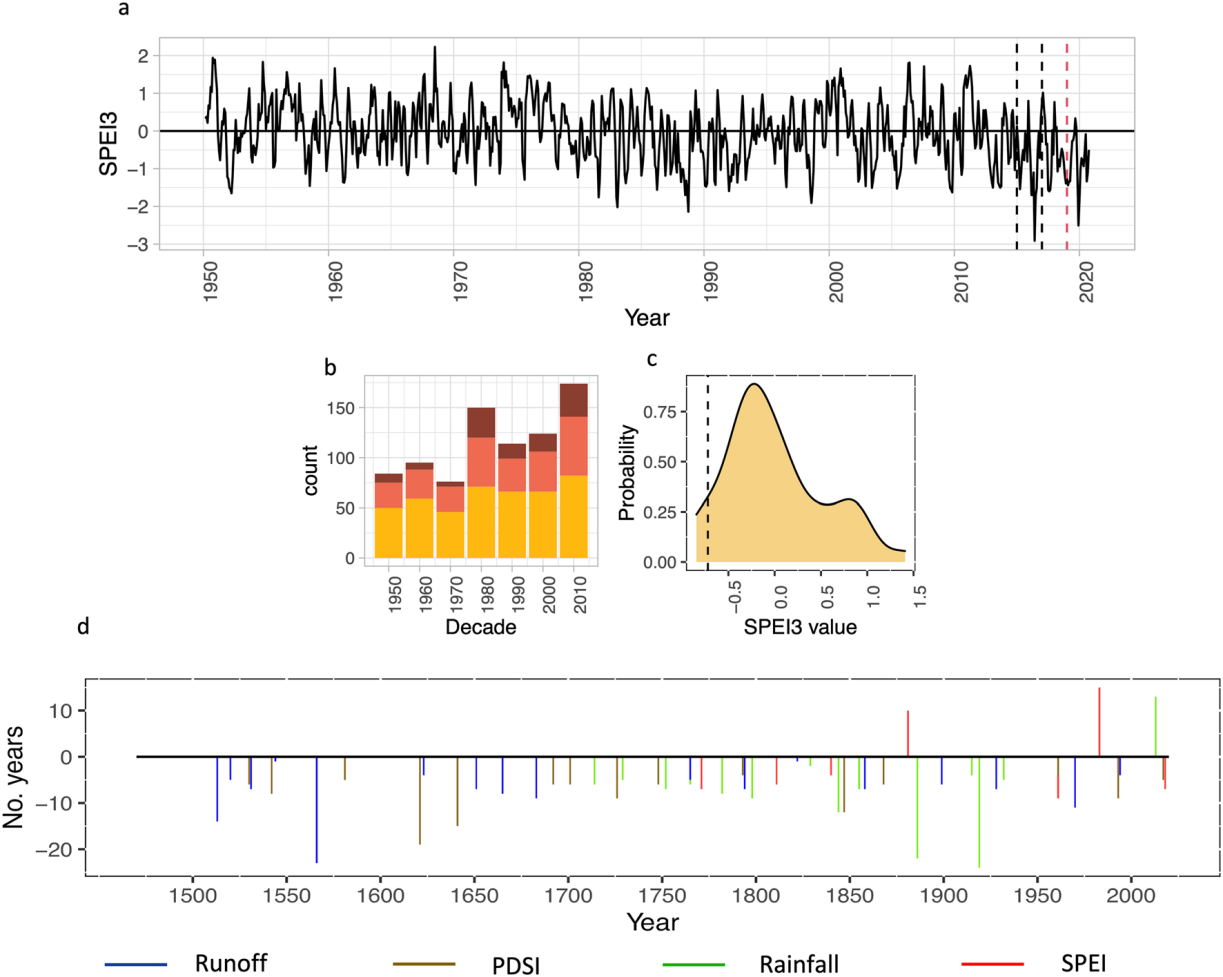
Hydroclimate and native tree deaths. (**a**) Monthly mSPEI3 values for the monsoonal north (for the box**—**17.75S 128.25E–10.25S–142.75E) from 1950 to 2020 (data source: https://spei.csic.es/database.html). Black dotted lines show the period from January 2015 to December 2016. Lowest mSPEI3 value recorded in June 2016. Red dotted line shows start of 2019. (**b**) Number of months per decade for which mSPEI3 < 0 (yellow), mSPEI3 < − 0.5 (coral), mSPEI3 < − 1 (dark red). (**c**) Distribution of 6-year non-overlapping averages of the March–May SPEI3 (reconstruction + instrumental). Dashed line shows the average SPEI3 value for the 2014–2019 period. (**d**) Runs of wet and dry years consistent with criteria described in main text according to the four different hydroclimate indices. A run of dry years is defined as at least 4 of 5 consecutive years with a value of − 0.5 s below the mean for the 1961–90 reference period. Longer dry periods may include up to two non-event years. Wet events include at least 10 wet years (+ 0.5 s) over a period of no more than 12 years. Y-axis indicates number of years events persisted. Negative values indicate extended dry events, positive values reflect extended wet events according to the criteria applied (Methods).

Notably, the mangrove dieback occurred during moderate to below average moisture conditions after almost three relatively wet decades (preconditioning event). These decades of above-average moisture conditions combined with increasing sea level over the same period effectively increased water availability to the mangrove ecosystem^9^, leading to a regional expansion of mangroves across much of northern Australia^7,9,10^. For *Avicennia marina* the increased water availability over the preceding decades facilitated a landward intrusion (sampled mangroves that died in 2015–16 in the Gulf of Carpentaria were 30–40 years old)^10,11^ and hence higher tidal elevation^6,9^ that left them vulnerable to a subsequent dry period, sudden fall in sea level, or both. Increased water supply to plants almost always results in an increased ratio of leaf to root area^12^, and mangroves with increased leaf to root area ratios are more vulnerable to drought stress^13^. The subsequent joint occurrence of reduced rainfall and a fall in sea level, lead to decreased water availability and acute physiological stress^11,14–16^ for the regional mangrove ecosystem. These stresses were driven by the effects of the 2015–16 Indian Ocean Dipole (IOD) and a strong El Niño event^11,17,18^ (Figs. 1, 2). Although precipitation was only slightly below average (Figs. 2, 3), low runoff levels for the Alligator River in Kakadu (Figs. 2, 3) may reflect the lack of tropical cyclone activity in 2015–16 which resulted in few intense rainfall events. Drought conditions, as represented by the Standardised Precipitation Evapotranspiration Index (SPEI) had also persisted for several years (Figs. 2, 3, 4) and temperature had been sharply rising for over 20 years.

**﻿Figure 1:**
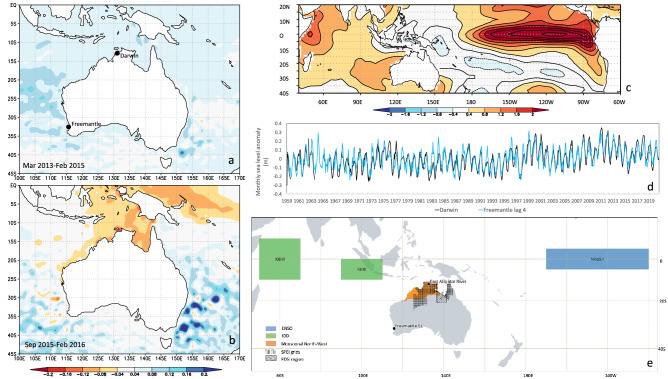
(**a**) Average sea level anomalies March 2013–February 2015; (**b**) Average sea level anomalies, September 2015–February 2016. Base period for these anomalies is 1993–2012 (see https://www.aviso.altimetry.fr/en/data/products/sea-surface-height-products/global/gridded-sea-level-anomalies-mean-and-climatology.html); (**c**) SST anomalies Sep 2015–Mar 2016. (**d**) Sea level data from Freemantle and Darwin showing very close relationship between the two; (**e**) Location of land-based palaeo-proxy records used in this study and climate model indices as well as the Niño 3.4 box and the east and west poles of the IOD index. Figure created using QGIS 3.10.

Similarly, the widespread dieback of inland trees across the Northern Territory was associated with a discrete shift from a multi-decadal period of wetter than average conditions to a period of intense drought. Since 2013, 80% of months experienced negative SPEI (3-month window) values and 61% had SPEI values below − 0.5 (Fig. 4a,b). In addition, the two most extreme negative monthly SPEI values since 1950 occurred in June 2016 (− 2.91) and December 2019 (− 2.50). SPEI values < − 2.0 reflect an extreme deficit in the water balance. The shallow soils in the worst affected areas are believed to have exacerbated reduced moisture availability (https://www.abc.net.au/news/rural/2020-11-13/widespread-tree-deaths-reported-on-nt-rangelands/12880710), and other factors such as land management practices cannot be ruled out.

Dieback events such as these have profound and extensive consequences for a range of ecosystem values across Australia’s Northern Territory. They can accelerate shifts in species composition, changes in forest structure, and rates of carbon loss and sequestration, as well as alter habitat availability for associated fauna and flora communities. However, it is unclear how unusual these events are. While the recent events have been widely reported as unprecedented, this primarily reflects temporal limitations of historical records and instrumental data (≤ 100 years), and standard sources describing forest health such as satellite imagery and forest inventory data (~  40 years), rather than rarity of CCEs. The shortness of these records provides few insights into the frequency and intensity of their environmental impacts.

To better understand CCEs, and their ecological impacts, we need longer records. Palaeoclimate proxies, such as tree rings, corals, and speleothems, have been widely used to reconstruct past climate. They can extend the instrumental climate record by centuries, offering greater insights into the nature of CCEs. As such, they provide a window onto the impacts of event sequence and/or co-occurrence on natural systems^7^. Here we use a range of proxy records describing sea level, hydroclimate and two broadscale modes of variability also linked to sea level variability (El Niño-Southern Oscillation (ENSO) and the IOD) to ascertain whether similar CCEs are likely to have previously occurred in this region. We first examine the probability of individual climate variables equalling or exceeding their respective 2015–16 magnitudes over both the instrumental and pre-instrumental periods. We then focus on the sequenced (pre-conditioning) and multivariate CCEs identified in the palaeoclimate compilation and use these inform our understanding of current events.

## Results and discussion

### Empirical probabilities of events in individual records

The probability of each individual variable recording a value at least as extreme as 2015 based on the full empirical probability distributions are generally comparable for the instrumental and palaeoclimate data (Fig. 2). The GEV probabilities (see Methods) are consistent with these estimates. The 25% difference between the instrumental and reconstructed precipitation reflects generally wetter conditions over the past ~ 30 years in the instrumental record (Fig. 2). For any one parameter, probabilities of events at least as extreme as those in 2015 were relatively, but not exceptionally, low in most instances (*p* < 0.1; Fig. 2). Neither precipitation nor the Palmer Drought Severity Index (PDSI) for 2015 were low, however (Figs. 2, 3).

With the exception of Nino 3.4 (one index of ENSO activity), none of the individual probabilities indicated exceptionally rare events (i.e. *p* > 0.01). This highlights the inadequacy of investigating the probabilities of isolated drivers^19^. Ultimately, modelling the complexity of the ecosystem impacts of CCEs requires flexible and sophisticated statistical models with the ability to incorporate multiple types of CCEs, such as, for example, multivariate and preconditioning events. Hidden Markov Models^20^ for example, are widely used to model sequenced events but rely on temporal independence**.** Our use of a 10-year moving average (Methods) violates this principle. Copulas are increasingly popular for modelling multivariate distributions, but their use in relation to the events described here is complicated by the importance of sequence, and how that sequence is defined. A deep dive into sophisticated statistical techniques is well beyond the scope of the current work. We instead focus on outlining periods identified in the compilation of records that are consistent with conditions in 2015–16 and 2020 to demonstrate the value of longer time series^19^ when considering CCEs and their environmental impacts.

### Mangrove dieback

A number of previous mangrove dieback events driven by various mechanisms have been documented^21^. Several, including the relatively small 2002–03 West Australia event (Table S1), have been linked with ENSO-related extremes^22–24^. The low canopy levels across northern Australia in 1987 that progressively recovered, stabilising after 2000, is compelling evidence of another undocumented dieback event, possibly associated with the very strong 1982–83 El Niño^6^. The climate data show a wet 1970s followed by drier conditions in the early 1980s (Figs. 2, 3), supporting the thesis that extended event sequences (in this case vegetative growth and expanding canopy followed by dieback) fundamentally impact environmental outcome^24^.

Analogously wet decades followed by a sharp deterioration in moisture conditions (a ≥ 1σ fall compared to the average of the previous 10 years) and a rapid decrease in sea level coinciding with El Niño, a positive IOD event or both have also occurred repeatedly in the instrumental records. Such instances include the 1940s–1950s, 1900s–1910s, and 1890s–1900s (Fig. 2).

According to the palaeoclimate records, similar sequences and joint anomalies (i.e. extended wetter periods followed by an abrupt fall in sea level and the onset of drought conditions) conditions occurred multiple times per century prior to 1900: twice in 1500s, four times in the 1600s, three times in the 1700s, five times in the 1800s and four times in the 1900s. The direct reconstruction of sea level (from 1795) indicates complex CCEs comprised of both joint and preconditioned events occurred in the 1880s, 1860s, 1840s and 1790s. Using the longer IOD and El Niño reconstructions as indicators of sea level variability^17,18^, the data indicate that a decade of wetter conditions followed by a rapid transition to dry conditions and fall in sea level occurred in the 1760s, 1740s, 1710s, 1650–60, 1640s, 1620s–30s, 1600–1610, 1580s and 1530s–40 (Fig. 2). Dry conditions marking the sudden change may have been shorter- (e.g. the 1860s event) or longer-lived (e.g. the 1830s). Despite the phase locking of ENSO and the IOD, the transition to drier conditions is more consistently aligned with Niño3.4 than the IOD (Fig. 2). There is approximately a 67% chance that lower sea levels co-occur with an El Niño event (≥ 1 s above the 1961–90 baseline) over the instrumental data period (1961–2019). This falls to 27% for the reconstructions (1795–1960). In contrast, co-occurrence of low sea level and a strong IOD (≥ 1 s above the baseline) event is 33% over the instrumental period, and 15% over the reconstructed period in common.

Our data also suggest there are extended periods for which CCEs similar to that in 2015–16 did not occur. These periods include the late 1600s–early 1700s, and much of the first quarter of the nineteenth and twentieth centuries respectively, and demonstrate variability over time in the occurrence of the type of CCE described here.

Overall, our data indicates that CCEs, of the nature described here, have likely resulted in repeated, and relatively frequent cycles of renewal, expansion and collapse. This pattern has persisted over multi-century time frames and is not particularly unusual in the context of the past 500 years (Fig. 2). Based on our data, the average return interval of such an event in this region is ~ 27 years. However, the rate of sea level rise is unprecedented^25^ over the past 220 years (Fig. 2), and as a fundamental driver of mangrove distribution^6^ is likely to significantly alter dynamics that have existed in the past.

Global Climate Model (GCM) simulations of future sea level trends agree that relative sea level will continue to increase^26^ and be superimposed on ENSO variability, which may itself be enhanced^27^, possibly resulting in more extreme droughts^28^. In addition, GCM projections indicate that tropical cyclones will become more intense, but less frequent^29^. This increasingly dynamic environment will likely further increase the vulnerability of northern Australian mangroves to future large-scale dieback events from multiple factors, some of which by themselves may not be extreme^19^. In the medium to long term, a more volatile environment may also limit expansion of mangrove habitat. The 2015–16 event may simply be a segue into a changing renewal, expansion and collapse regime likely to lead to changes in distributions amongst mangrove species in the future^9^.

### Inland forest and savanna dieback

Eucalypt dieback has been a recurrent theme across much of Australia for at least 70–80 years, and in rural areas has been widely attributed to insect herbivory and/or land management issues^30,31^. Recent work, however, has identified strong links with severe drought conditions^32^.

The monthly SPEI3 data (Fig. 4a) for the region in which dieback has occurred reveals the persistence of the dry conditions since 2013 and a general trend towards a higher proportion of dry months (i.e., negative SPEI3) since 1950 (Fig. 4b). Additionally, low SPEI3 over the 2013–2019 period as a whole is unusual, although not unprecedented according to the annual March–May reconstruction, with 1947–1952 and 1838–1844 being, on average, somewhat drier (Fig. 4c). However, we find no evidence over the past 500 years for decadal or longer wetting periods immediately followed by a dry period of > 5 years. The two hydroclimate reconstructions that extend before 1700, especially the runoff reconstruction, do suggest wetter conditions existed in the late 1500s followed by a generally drier period that persisted until 1650. Aside from the most recent wet period, two other extended wet periods can be inferred from the data; in the 1970s and the 1870s (Fig. 4d). Neither of these periods, however, met our criteria for defining an event because they were not immediately followed by a sufficiently long dry period (≥ 5 years).

In the context of the applied criteria and a multi-century record, the duration and intensity of the most recent dry period since 2013, (Figs. 2, 4c) following nearly three decades of wetter than average conditions, appears unusual. The lack of analogous events over the past ~ 500 years suggests that the inland forests and savannas are likely being subjected to levels of climatic and physiological stress that are unprecedented over the past half millennium. Interestingly, if thickening and expansion of vegetation cover is tightly coupled with moisture availability, the data suggest vegetation thickening in the region is likely to have occurred only over the past 150 years (Fig. 4d). If, however, it were possible to ignore the importance of event sequence and inland savanna tree dieback was solely predicated on protracted drought events (≥ 5 years), the hydroclimate data suggests that previous dieback events have likely occurred between two and four times per century over the past 500 years (Fig. 4d; return interval varies depending on reconstruction). This would suggest that widespread dieback events across the savanna are not particularly rare.

Relatively persistent increases in temperature since the latter part of the twentieth century and projections of continued increase, as well as an increased number of days with temperatures > 35 °C^33^ may worsen local drought conditions by increasing evaporative demand. Projections that indicate increased aridity in eastern Australia will be primarily driven by increasing temperature^34^ are particularly concerning. Without a locally relevant high-resolution temperature reconstruction available for the past five centuries, it is not possible to assess how temperature has covaried with previous wet and dry periods revealed in the compiled palaeoclimate records. If sequencing of events plays a critical role in dieback on the scale of that in 2020, increased aridity alone does not infer increasing frequency of dieback events. A continuation or otherwise of the ~ 20-year trend towards increased rainfall variability^33^ will play an important role in regulating tree and shrub establishment. But low confidence in hydroclimate projections for the coming century^33^ make it difficult to even speculate as to whether increases in woody growth that can subsequently become susceptible to sustained drought activity is likely to increase. Increased aridity cannot kill vegetation that never established.

### Caveats/limitations

The 2015–16 mangrove dieback in the Gulf of Carpentaria primarily affected *Avicennia marina*, a species typical of landward mangrove communities, and our focus has been on conditions relevant to that event. However, causes of mangrove dieback are complex, and include cyclones, tsunamis and insect predation^6^^,^^11,35^, all of which are impacted by climate variability. For the more seaward mangroves such as *Sonneratia alba* and *Camptostemon schultzii* other factors such as cyclone activity may be more important in shaping forest dynamics. Palaeo-storm reconstruction would provide greater insight into past variability in cyclone activity and potential impacts of storms on seaward mangroves^35,36^. Factors such as tidal regime^9^ and wave energy^37^ are also likely to be relevant: the far north of Australia experiences a diurnal mesotidal regime with low wave energy which plays an important role in sediment dynamics and mangrove distribution. Additionally, phenological differences by site and species^35,38^ that mediate the impact of climate extremes and non-climatic factors (e.g., geomorphological factors such as soil depth) may also be relevant. Our use of an average mangrove forest recovery time of 10 years may not represent all mangrove forests, as recovery time will be dependent on localised environmental factors. Similarly, while our criteria for identifying past inland savanna tree dieback were consistent with observed climate leading up to the 2020 dieback, different criteria would likely have produced slightly different results. Additionally, consideration of the nexus of other environmental factors in conjunction with climatic conditions that lead to major dieback events may produce different results, but additional extensive data would be required to assess this.

In terms of broadscale climate variability, we have included only two important modes in our study, the IOD and ENSO. However, more transient modes, such as the Madden–Julian Oscillation (MJO) which exhibits a strong relationship with sea level and rainfall bursts in the Gulf of Carpentaria^39^, also play a key role in northern Australian hydroclimate^40,41^. Our investigation of the climate data has also been limited to particular seasons covered by the annually resolved palaeoclimate reconstructions. Although this may limit the extent of inference regarding CCEs that lead to the observed impacts, the tropics are highly seasonal and previous work has indicated that the hydroclimate reconstructions largely capture changes in interannual wet season variability^42^. In addition, the IOD and ENSO are subject to seasonal phase-locking.

No palaeoclimate record is a perfect representation of a climate variable because each reflects the state of a multi-facetted environment. Uncertainty in the reconstructions is readily apparent from a comparison with respective instrumental time series (Fig. 3a). The different hydroclimate reconstructions also detect slightly different mixes of events (Fig. 3b), this being associated with differences in seasonal target. Only a handful of events are detected by all four hydroclimate reconstructions. Statistics for each reconstruction used in this study (Fig. 3a) provide measures of uncertainty for each but equally demonstrate that each reconstruction provides valuable insight into past conditions. Nevertheless, it is relevant to consider some sources of uncertainty in the compiled reconstructions. Firstly, the sole (SPEI) or heavy (PDSI, precipitation, runoff) reliance on tree-rings may have resulted in a bias towards representation of dry rather than wet conditions. Tree-rings typically reflect dry conditions better than wet conditions because moisture is more limiting under dry conditions^43^. Palaeo-hydrological records derived from independent sources would likely improve the detection of extended wet periods that may have existed over the past 500 years. Secondly, temporal uncertainty can also exist where records are not absolutely dated (e.g. see Table S2 in Abram et al. 2020^44^). Thirdly, for reconstructions relying heavily on remote proxies^45,46^, changing strength of teleconnections may also contribute to uncertainty. Our compilation of multiple records is an explicit recognition that no one palaeoclimate record will register all aspects of a hydrological event with extreme environmental impacts. Similarly, by focusing on general consistency amongst records we recognise the limitations and subtle differences in the various reconstructions.

Finally, identifying the relevant aspects of CCEs and their interaction with other biophysical factors that lead to a major environmental impact is crucial to informing our understanding of past impacts and how climate change is affecting the nature of CCEs impacting ecosystems. Ignoring a critical element (e.g. the importance of event sequence for inland savanna dieback) may lead to erroneous conclusions as to the rarity of such events.

## Concluding comments

Compound climate extremes can act as regional-scale disturbances that threaten ecosystem services, such as carbon storage and habitat provision. However, these events are underrepresented in instrumental data sources and therefore poorly understood. We have shown that by drawing together a diverse range of palaeoclimate records, it is possible to significantly extend the record of likely CCE occurrence to place current extreme impacts into the appropriate historical context. While the climatic conditions associated with the mangrove dieback have occurred with several times per century over the past 500 years, the interaction with rapidly increasing sea level is unique over the past 220 years. At the same time, the nature of the CCE associated with the dieback of inland savanna forests appears to be without precedent over the past 500 years. However, both these conclusions are dependent on the criteria used to identify events. By utilising a compilation of locally relevant records, we have ameliorated some of the inherent uncertainty contained in individual palaeoclimate records. Nevertheless, additional independent sources of evidence for historical dieback events, whether through palaeoecological proxy records (e.g., pollen analysis, sediment cores, dendrochronology and or biophysical modelling) or through the knowledge of the Traditional Owners would help to unravel the consequences of CCEs on these ecosystems at time scales commensurate with palaeoclimate proxies. We contend that our results demonstrate the value of an increased emphasis on the use of long palaeoclimate records in CCE research. This is especially the case when extended event sequences and complex CCEs result in lasting impacts on ecosystems and environments. Coupling of deep understanding of ecosystem response to climate and biophysical variability with the long term palaeoclimate records is needed to better understand past, present and changing environmental impacts of CCEs. These long records will inform our understanding of how risk profiles of how damaging environmental events are changing, and the nature of ecological resilience to past occurrences.

## Materials and methods

To better understand how the identified factors have covaried over long time frames, we compiled palaeoclimate reconstructions of target variables previously associated with the dieback events (Table 1). These include fluctuating sea level (mangrove event), changing moisture availability (mangrove and inland savanna events), drought (mangrove and inland savanna events), and the ENSO and IOD states (mangrove event (sea level and drought impacts) and inland savanna dieback (drought impacts)).

**Table 1:** Data used.

Record	Reconstructed period	Instrumental data period	Season (months)	References
Indian Ocean Dipole (IOD)	1241–1869*	1870–2019	JASOND	Abram et al. 2020, downloaded from https://psl.noaa.gov/gcos_wgsp/Timeseries/Data/dmi.had.long.data
Nino3.4	1300–1869	1870–2019	NDJ	Li et al. 2013; instrumental period record downloaded from https://psl.noaa.gov/gcos_wgsp/Timeseries/Data/nino34.long.data
Sea level	1795–1959	1959–2019 (some gaps filled with reconstructedvalues)	Jan–Dec	Zinke et al. (2014); see Fig. 1 for comparison with Darwin record
Palmer Drought Severity Index (PDSI)	1500–1900	1901–2017	DJF	Palmer et al. 2015; https://crudata.uea.ac.uk/cru/data/drought/
Standardised Precipitation Evaporation Index (SPEI3)	1761–1900	1901–2019	MAM	Allen et al. 2020; instrumental period record downloaded from https://spei.csic.es/database.html
mSPEI3	NA	1950–2019	Monthly data	Instrumental period record downloaded from https://spei.csic.es/database.html
Temperature	NA	1901–2019	ONDJFM	Instrumental period record downloaded from https://spei.csic.es/database.html
Precipitation	1708–1900	1901–2017	ONDJFM	Freund et al. 2017; instrumental record downloaded from https://dataplatform.knmi.nl/catalog/index.html
Runoff	1470–1976	1977–2019 +	Sep–Aug	Verdon-Kidd et al. 2017

*Reconstruction does not cover entire period. + Simulated runoff based on precipitation at Oenpelli. No regionally specific high-resolution temperature reconstruction is available.

As a fundamental physical factor for mangrove habitat^6^, changes in sea level, whether positive or negative, will affect populations and species composition**.** A fall in sea level can deleteriously affect mangrove populations reliant on tidal inundation, especially when coupled with high temperatures and extended drought conditions^6,12^. We therefore sourced an annually resolved Fremantle sea level reconstruction extends back to 1795 CE^25^ (Table 1) that is highly correlated with Darwin sea levels (r = 0.77, Fig. 1d), providing a reasonable surrogate in the absence of an in-situ sea level reconstruction.

Impacts of the tightly coupled Pacific and Indian Ocean variability^47^ on Australia’s hydroclimate are significant. A positive IOD event is typically associated with drier winter–spring conditions from northwestern to southeastern Australia^48^. ENSO’s widespread impact on Australian hydroclimate, including that on the Northern Territory, is well documented^33,48,49^ and recurrent El Niño activity has been associated with the severe rainfall deficit across eastern and northern Australia during one of Australia’s most severe droughts of the twentieth century, the Federation drought^49^. The Settlement Drought of the early 1790s that severely impacted the early settlements, native wildlife, crops and vegetation in southeastern Australia has also been attributed to a period of sustained ENSO activity^50,51^. Sea level falls are also commonly associated with El Niño events^19^. Due to the widespread and significant impacts of these major climate modes, we include an ENSO reconstruction that extends back to 1301 CE^52^, and a discontinuous IOD reconstruction^52^ that covers approximately half of the last millennium in our compilation of palaeoclimate reconstructions (Table 1). Although the hydroclimate impact of the IOD across northern Australia once the effect of ENSO is removed is relatively small^48^, the IOD is retained in our data set due to its links with sea level variability^17^. Instrumental indices of ENSO and the IOD extend back to 1870 (Fig. 3). We examined both the Niño3.4 and IOD indices and reconstructions for positive events associated with decreased sea level and/or dry conditions in northern and eastern Australia.

There are four locally relevant hydroclimate reconstructions for the region, each coupled with a set of instrumental data. The first reconstruction is a warm season precipitation reconstruction for monsoonal Australia^45^ that extends back to 1708 CE. Instrumental precipitation data utilised covers the same spatial area as the palaeoclimate record and is drawn from the gridded Australian Water Availability Project (AWAP) precipitation data (see Fig. 1e). Two gridded drought index reconstructions based on the Standardised Precipitation Evaporation Index (SPEI3; Mar–May)^42^ and the Palmer Drought Severity Index (PDSI; Dec–Feb^51^) exist and extend back to 1762 and 1500 respectively. Like the reconstructed PDSI, instrumentally based PDSI data cover the area around the Gulf of Carpentaria across to Darwin (Fig. 1e). Two slightly different instrumental SPEI datasets include the annual March–May SPEI3 data and a shorter SPEI3 data set available at monthly resolution (mSPEI3) for the same region covered by the SPEI3 reconstruction^42^ (Fig. 1e). There is also a runoff reconstruction for the Alligator River^46^ (Table 1; Fig. 1e) that covers the period 1470–1976. This reconstruction is simulated from precipitation, and hence we use the precipitation as the relevant instrumental partner record (Table 1). In all cases, seasons included in our compilation of instrumental records were consistent with those available for the corresponding palaeoclimate record. There is no highly resolved temperature reconstruction specific to this region, but we include the instrumentally based AWAP maximum temperature record for the monsoonal north (same region as precipitation data; Fig. 1e).

With reference to climate conditions preceding and during the two events, we identified, in a semi-quantitative manner, similar sets of conditions in the past. The 2015–16 mangrove dieback event saw a rapid fall in sea level of  > 1 s compared to the average of the previous 10 years. Expansions and contractions of mangroves have previously occurred over relatively short periods^9^ and it has been reported that mangroves can recover over a period of around 10 years^15^. On the basis of this evidence and the observed event, we compared sudden falls of  ≥ 1 s in sea level relative to average sea level for the previous decade (based on a 10-year moving average) accompanied by indication of increased aridity (i.e. sudden increase of ≥ 1 s in aridity relative to conditions over the past decade, as indicated by a 10-year moving average) in the hydroclimate data (precipitation, simulated runoff and drought). The same criteria were applied to both the instrumental data and palaeoclimate reconstructions.

There is less information available concerning the very recent inland tree dieback across the Northern Territory. However, it has also occurred against a background of a multi-decadal wetting trend (and the resultant thickening of vegetation), followed by at least five years of drier than average conditions (runoff was below average for five years, SPEI for six). Therefore, we examined the palaeoclimate data for extended wetter periods (≥ 10 years within a 12-year period at least 0.5 s above the mean) followed by periods of dry conditions (using a threshold of at least 0.5 s below the mean) that persisted over at least four of five consecutive years.

When examining the palaeoclimate data, we searched for general consistency amongst the hydroclimate proxies rather than requiring all to capture the event. There are several reasons for this. Firstly, all palaeoclimate reconstructions contain some uncertainty around the estimate (Fig. 3) simply because they are affected by their micro-environment as well as the regional climate. Secondly, an extreme impact associated with a multivariate CCE can still occur even when individual climate variables are not extreme^3,19^. We have not attempted to use more sophisticated statistical techniques because these are limited for higher dimensional events (> 2 dimensions)^20^ and beyond the scope of this short contextual study. Crucially, the mangrove dieback in particular includes elements of both sequential and multivariate CCEs, further complicating statistical modelling of this dieback event. Additionally, long-term information on the occurrence of actual dieback events, which would be an important element of many of these quantitative analyses, is lacking. Further, our purpose here is to provide a simple example of how palaeoclimate proxy records might, in the first instance, be used to identify sequences and co-occurrence of specific conditions prior to the instrumental record period.

Although modelling the nature of the CCEs involved in these dieback events is complex and beyond the scope of this study, we estimated the empirical probability of each variable recording a value at least as extreme as that in 2015. We also estimated the probability based on a Generalised Extreme Event distribution focused on the relevant tail of the distribution (i.e. lower tail for precipitation, runoff, SPEI and PDSI, detrended sea level, and the upper tail for Nino3.4 and IOD). For each climate variable, these calculations were based on the ‘merged’ instrumental and palaeoclimate data. This merged data comprised the instrumental data as far back as it extended prior to which the relevant reconstruction data was used. This information serves to emphasise differences amongst various hydroclimate variables. The inability of these single factor probabilities to capture the probability associated with the sequenced and co-occurrence of multiple stress factors underlying the dieback events^9–11,14–16^, reiterates the complex nature of CCEs and their resultant impacts on ecosystems.

## Supplementary Information

Supplementary Table S1.
