# Supplementary material for: Compound climate extremes driving recent sub-continental tree mortality in northern Australia have no precedent in recent centuries
Source: Sci Rep. 2021 Sep 15;11:18337. doi: 10.1038/s41598-021-97762-x (PMC8443737; doi:10.1038/s41598-021-97762-x)
Supplement: Supplementary file 1 — Supplementary Table S1. [file 41598_2021_97762_MOESM1_ESM.pdf]

Supplementary material

Compound climate extremes driving recent sub-continental tree mortality in northern Australia have no precedent in recent centuries

Kathryn J Allen[1,2,3,*], Danielle C Verdon-Kidd[4], James Z Sippo[5], Patrick J Baker[2]

[1] School of Geography and Spatial Science, University of Tasmania, Sandy Bay AUSTRALIA 7005

[2] School of Ecosystem and Forest Sciences, University of Melbourne, Richmond AUSTRALIA 3121

[3] ARC Centre for Australian Biodiversity and Heritage, UNSW Node, AUSTRALIA

[4] School of Environmental and Life Sciences, University of Newcastle, Callaghan AUSTRALIA 2308

[5] Faculty of Science and Engineering, Southern Cross University, Lismore AUSTRALIA 2480

* Corresponding author: Kathryn Allen

Email: Kathryn.Allen@utas.edu.au

Year	Region	Cause	Area (ha)
1971	Townsville, Queensland	Cyclone Althea/siltation	~5
1980	North Queensland	Pathogen	Patches
1994	Gladstone Queensland	Hail storm	0.1
1999	Exmouth, Western Australia	Cyclone Vance	5700
2002-3	Exmouth	ENSO-related climate extremes	40
2011	Hinchinbrook Island, Queensland	Cyclone Yasi	2200
2015	Gulf of Carpentaria, and across other parts of northern Australia, Exmouth, Western Australia	ENSO-related climate extremes	~7400 ha in Gulf of Carpentaria, extensive elsewhere

Table S1. Other mangrove dieback events recorded for Australia. Source: Sippo et al. 2018
